# Spatially resolved and orientation dependent Raman mapping of epitaxial lateral overgrowth nonpolar a-plane GaN on r-plane sapphire

**DOI:** 10.1038/srep19955

**Published:** 2016-01-29

**Authors:** Teng Jiang, Sheng-rui Xu, Jin-cheng Zhang, Yong Xie, Yue Hao

**Affiliations:** 1 Wide Bandap Semiconductor Technology Disciplines State Key Laboratory, School of Microelectronics, Xidian University, Xi’an 710071, China; 2 School of Advanced Materials and Nanotechnology, Xidian University, Xi’an 710071, China

## Abstract

Uncoalesced a-plane GaN epitaxial lateral overgrowth (ELO) structures have been synthesized along two mask stripe orientations on a-plane GaN template by MOCVD. The morphology of two ELO GaN structures is performed by Scanning electronic microscopy. The anisotropy of crystalline quality and stress are investigated by micro-Raman spectroscopy. According to the Raman mapping spectra, the variations on the intensity, peak shift and the full width at half maximum (FWHM) of GaN E_2_ (high) peak indicate that the crystalline quality improvement occurs in the window region of the GaN stripes along [0001], which is caused by the dislocations bending towards the sidewalls. Conversely, the wing regions have better quality with less stress as the dislocations propagated upwards when the GaN stripes are along [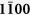]. Spatial cathodoluminescence mapping results further support the explanation for the different dislocation growth mechanisms in the ELO processes with two different mask stripe orientations.

As a typical representative of the third generation of semiconductor materials, gallium nitride (GaN) has outstanding properties with a wide band gap of 3.42 eV. It is a very attractive material especially for application in electronic and optoelectronic devices. The majority of commercial GaN devices are grown with respect to the c-plane. The polarization field along c-axis could give rise to a significant band bending and thus cause the separation of holes and electrons which is beneficial to the High Electron Mobility Transistor (HEMT) devices, while this situation could also induce the reduction of optical efficiency of light-emitting diodes (LEDs), as well as an undesirable redshift in the emission spectra from the quantum wells what is called the quantum-confined Stark effect (QCSE)^1^. Detailed theoretical calculations and experimental results have shown that the QCSE is smaller in quantum dots than in quantum wells^2^,^3^,^4^,^5^,^6^. Many researches inhibited the QCSE by using strain-free nanocolumn^7^, nanorod arrays^8^,^9^ and other 3D nanostructures^10^,^11^. In addition, one of the most promising approaches for avoiding the negative effects of the QCSE is that GaN epilayers should be grown along nonpolar orientation such as (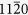) a-plane. However, there exist very high extended defect densities in a-plane GaN, including stacking faults and threading dislocations (TDs) with the density of 10^5^–10^6^ cm^−1^ and 10^9^–10^10^ cm^−2^ respectively^12^,^13^,^14^,^15^, and very serious anisotropy^16^,^17^,^18^,^19^, which could have a great influence to the emission efficiency. The technique of epitaxial lateral overgrowth (ELO) is an effective method to reduce dislocations and enhance material quality^20^,^21^,^22^,^23^. Several approaches for reducing the dislocation density of a-plane GaN have been demonstrated by ELO^24^,^25^. Structural and optical properties of ELO GaN grown on r-plane sapphire substrate have been investigated^26^,^27^, however, the influence of anisotropy in nonpolar ELO GaN on the variations of dislocations and stress along different mask stripe directions is rarely studied. To observe the growth mechanism and the anisotropy of GaN along different orientations, we stopped the process before the coalescence of the GaN films. As a sensitive and non-invasive, non-contact optical testing method, Raman spectroscopy is a widely used method in research of lattice properties, crystalline quality and stress distributions of semiconductor materials. In this study, we demonstrate spatially resolved micro-Raman scattering results for mapping the spatial variations in crystalline quality and stress in the ELO a-plane GaN structures grown along different mask stripe orientations.

## Experimental

**Figure 1:**
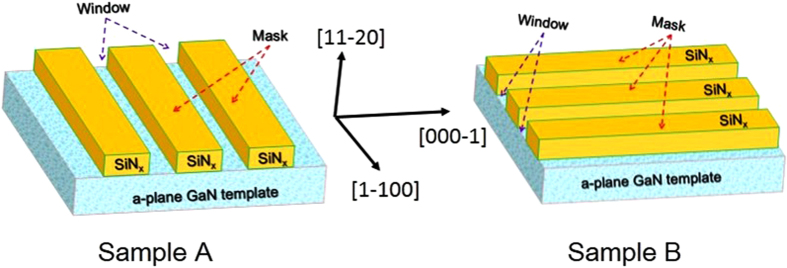
Schematic diagram of the masks along different orientations in sample A and B.

A 1.2 μm thickness a-plane GaN template with a low temperature AlN buffer layer (20 nm, 620 °C) was grown on an r-plane sapphire substrate using homemade metal organic chemical vapor deposition system. Triethylgallium (TEG) and ammonia (NH_3_) were used as source compounds. Hydrogen was used as the carrier gas. The growth temperature was kept at 1000 °C. A 100-nm-thick SiN_x_ film was subsequently deposited onto the a-plane GaN template. Then, as shown in Fig. 1, the SiN_x_ film was patterned into 5-μm-wide mask stripes and 2-μm-wide window stripes oriented along the [0001] and [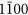] direction respectively, using a standard photolithography technique. Next, the ELO a-plane GaN was regrown using two step (1040 °C and 1080 °C) method in order to encourage lateral overgrowth. The growth was stopped before the GaN stripes coalesced so that the growth details and the anisotropy of crystal quality and stress can be better studied. Here we name the sample grown on the mask stripe oriented along [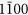] as sample A, and the other as sample B. After the regrowth, the morphology of the samples was investigated by Scanning electron microscopy (SEM). The Raman spectra were measured at room temperature using a confocal Jobin Yvon LavRam HR800 micro-Raman spectrometer with a charge-coupled device (CCD) detector and an optical microscopy system. The wavelength of the excitation laser was 514 nm and the laser power was about 2 mW. The laser beam was focused to a spot with a diameter of about 2 μm on the sample surface using a 50× microscope objective. All Raman spectra were measured in x (y, y) 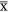 backscattering geometries. Here we defined z‖[0001], x‖[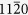], and y‖[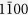]. An automatic XY stage allowed the samples to be moved in small steps of 0.1 μm. Cathodoluminescence (CL) analysis was performed using an FEI Quanta 400 FEG operating at 20 kV and equipped with a Gatan MonoCL3+ system.

## Results and Discussion

**Figure 2:**
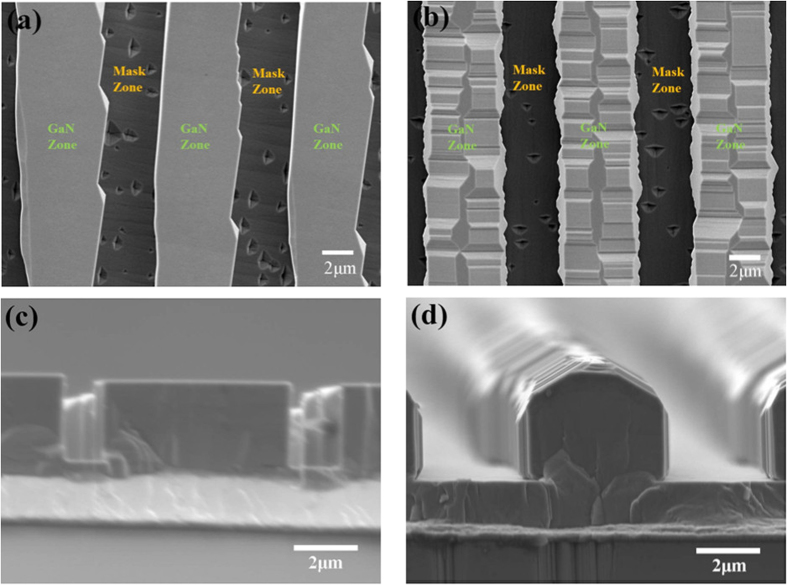
(**a,b**) Top view SEM images of uncoalesced ELO GaN samples: (**a**) sample A and (**b**) sample B; (**c,d**) cross-sectional SEM images of samples: (**c**) sample A and (**d**) sample B.

To investigate the morphology of the uncoalesced ELO GaN stripes along different orientations, both the samples have been measured by SEM. Figure 2 shows SEM images of both the samples. Triangle pits appear at the template surface of uncoalesced regions in both samples. As a typical characteristic, it has been reported that these triangular pits were composed of one [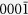] and two [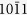] facets^18^,^19^. So it further confirms that the stripes are along the [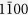] direction in sample A, and parallel to the [0001] direction in sample B. Also the effect of stripe orientation on the ELO morphology is clearly observed. As shown in Fig. 2(a), the ELO GaN stripes along the [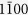] orientation are about 5 μm wide with flat top facets while the top facets of the stripes are almost vanished in sample B. Similar to other studies^26^,^28^,^29^, the [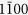] GaN stripes have rectangular cross-sections with vertical [0001] and [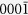] sidewalls as shown in Fig. 2(c). In contrast, the GaN stripes along [0001] have a polygon cross section with inclined facets both from the same crystallographic family of [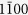] planes^28^ and from the same crystallographic family of [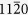] planes. However, further study is needed to clarify the more detailed relationship between the stripe orientation and the crystallographic plane of the sidewalls. The cross-section SEM images also prove that the vertical growth rate in sample B is faster that of sample A while the lateral overgrowth rate of sample B is slower under this growth condition. In other words, the ELO GaN stripes along the [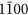] needed less growth time to coalesce with thinner thickness. The morphology variation of these samples indicates that the crystalline quality and stress distribution may have anisotropy with respect to the dislocation spatial variations along different directions.

**Figure 3:**
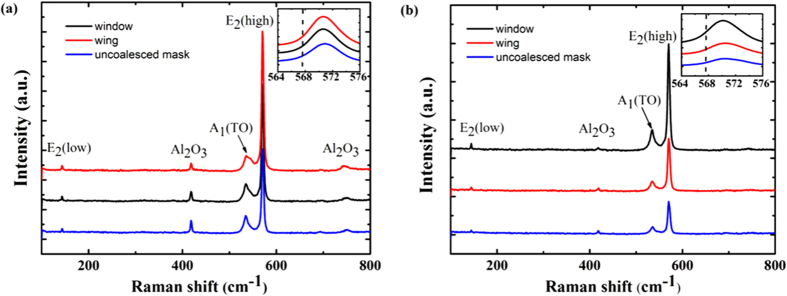
Representative Raman spectra obtained from wing, window and uncoalesced mask regions of both samples. (**a**) sample A and (**b**) sample B. Insets illustrate the magnification of the E_2_ (high) mode peaks of GaN.

In order to investigate the spatial variation in stress and crystal quality in the ELO GaN structures along different stripe orientations, micro-Raman measurements have been carried out for both ELO GaN samples. According to group theory predicts^30^, taking hexagonal GaN belonging to the 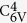 space group, there should be eight sets of phonon modes near k = 0: 2A_1_ + 2E_1_ + 2B_1_ + 2E_2_, among which one set of A_1_ and E_1_ mode are acoustic and two B_1_ modes are Raman inactive. Only one A_1_, one E_1_ and two E_2_ modes are Raman active. The A_1_ and E_1_ symmetries are split into longitudinal (LO) and transverse (TO) components. So there exists six first order Raman-active optical modes at Γ point: A_1_ (LO) + A_1_ (TO) + E_1_ (LO) + E_1_ (TO) + E_2_ (low) + E_2_ (high) in various scattering geometry configurations. Under x (y, y) 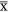 geometry, only E_2_ (low) A_1_ (TO) and E_2_ (high) mode peaks are allowed to appear in the spectra. The E_2_ (high) mode peak is considered to be the most sensitive to stress and always used to characterize the residual stress in GaN thin films^31^. The E_2_ (high) mode peak of strain-free GaN should be at 567.6 cm^−1^, while the peak would up shift when it is under compressive stress^32^. Figure 3 illustrates representative Raman spectra extracted from the wing, window and uncoalesced mask regions of both samples. As show in Fig. 3(a), three peaks located at about 142 cm^−1^, 537 cm^−1^ and 570 cm^−1^ appear in all spectra which represent to the E_2_ (low), A_1_ (TO) and E_2_ (high) mode peak respectively^33^. The peaks at about 418 cm^−1^and 748 cm^−1^ are sapphire Raman peaks^34^. It can be obviously noticed from the insets that all the E_2_ (high) mode peaks in the two samples are larger than 567.6 cm^−1^ which means that all the regions are under compressive stress, as predicted for the GaN grown on sapphire substrates^32^.

**Figure 4:**
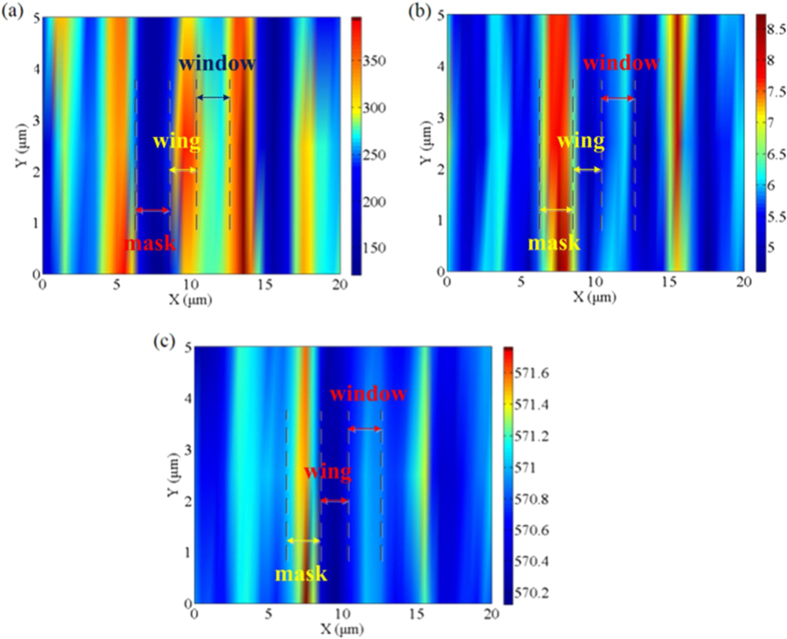
(**a**) Raman mapping spectrum of E_2_ (high) mode intensity of sample A; (**b**) Raman mapping spectrum of E_2_ (high) mode FWHM of sample A; (**c**) Raman mapping spectrum of E_2_ (high) mode peak position of sample A.

A spatial mapping of the E_2_ (high) mode provides useful information on spatial distribution of crystalline quality and stress in the ELO GaN structure. The mapping scan data are presented in a false-color image, with hues ranging from blue for the background to red for the highest intensity. To extract values for intensities, peak positions and the full width at half maximums (FWHMs), individual E_2_ (high) modes are fitted to a Lorentzian-Guassian mixed function. Figure 4(a) shows an intensity map of the E_2_ (high) mode in a 20 × 5 μm^2^ zone measured in sample A. The distinction in intensity between the wing, window and uncoalesced mask regions is easily distinguished. The intensity in the wing region is the strongest, indicating the perfect crystalline quality exists in this region. The window region has slightly weakened intensity which is higher than that of the mask region. In Fig. 4(b), the FWHM of E_2_ (high) mode in the window region is higher than that of the wing region which also proves that the crystalline quality in the wing region is better than in the window region. As shown in Fig. 4(c), the spatial stress has the similar variations to the FWHM of E_2_ (high) peaks. The minimum stress exists in wing regions while the maximum stress is in the uncoalesced regions. The stress in the window regions is relatively larger than that in the wing regions. The variation of E_2_ (high) mode frequency also indicates the stress should be with respect to the dislocations and defects. According to T. Kitamura *et al.*’s research^25^, the Raman peak frequencies increase with increase of dislocation density. So the reduction of dislocations in the wing regions may lead to the decrease of residual stress. (As shown in Fig. 1 in the supplementary information file, similar variations on E_2_ (high) peak of coalesced GaN layer between the window and wing regions are observed.)

**Figure 5:**
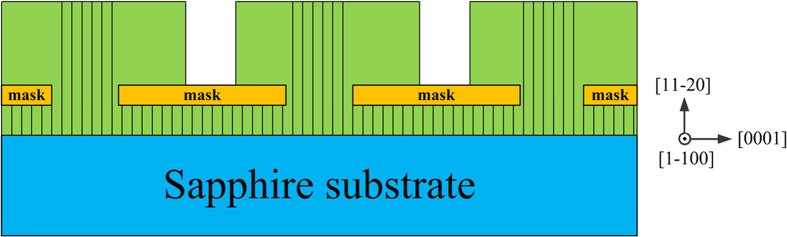
Schematic diagram of dislocation growth mechanism during GaN ELO process of sample A.

The variations of crystalline quality and stress distribution in different regions can be attributed to the dislocation growth mechanism during the lateral regrowth. As shown in Fig. 5, when the mask stripes are along the [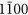] direction, the lateral growth directions which perpendicular to the stripes are Ga polar and N polar directions. Almost all the dislocations prefer to grow perpendicular to the substrate instead of tending to bending towards the c-plane^35^. The dislocations are stopped propagating upward when they reach the mask. Conversely, in the window regions, the dislocations grow up towards the top facets. So the dislocation density in the wing regions is lower than that in the window region, leading to the improvement of quality in the wing region and weaker residual stress.

**Figure 6:**
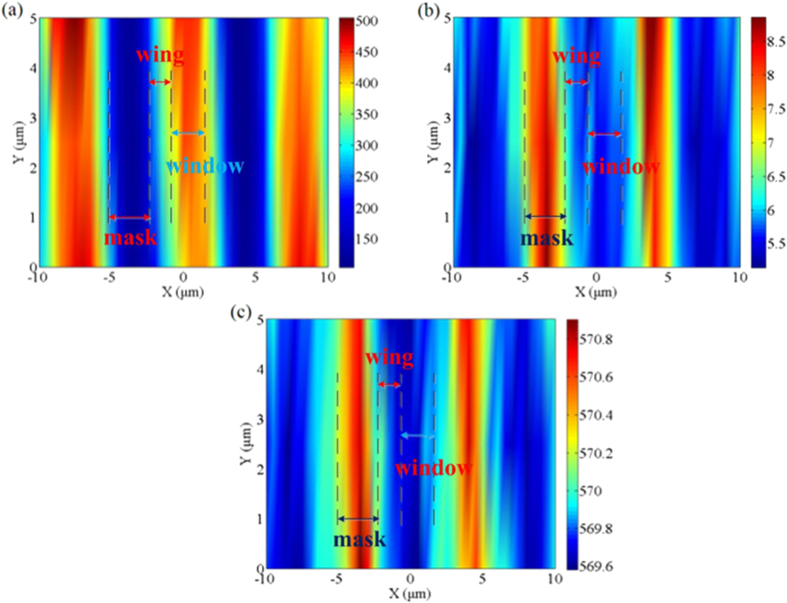
(**a**) Raman mapping spectrum of E_2_ (high) mode intensity of sample B; (**b**) Raman mapping spectrum of E_2_ (high) mode FWHM of sample B; (**c**) Raman mapping spectrum of E_2_ (high) mode peak position of sample B.

By comparison, Fig. 6 illustrates the Raman mapping spectra of sample B. There are obvious differences between the two samples on the intensity, peak shifts and FWHM of the E_2_ (high) mode. The intensity plot of the E_2_ (high) mode exhibits several distinctive features. Firstly, the intensity of E_2_ (high) in the wing region is higher than that in the uncoalesced region and smaller than in the window region. The intensity of the E_2_ (high) is stronger in the window regions by approximately 25% than that in the wing regions. The enhancement of E_2_ (high) intensity indicates that crystalline quality is better in the window regions than in the wing regions^36^. On the contrary, the variation trends of the E_2_ (high) mode FWHM in the wing and window regions are opposite to that of intensity in Fig. 6(b). The maximum width of E_2_ (high) spectral occurs at the mask region, which is in agreement with the poor quality of a-plane GaN template under the mask. These broadened FWHM of the wing region than that of the window region also consistent with the decrease in the Raman intensity in the GaN wings. Fig. 6(c) shows the spatial variation in peak value of E_2_ (high). It can be found that all the regions suffer from compressive stress for all the peaks shifted upward. The E_2_ (high) peak is gradually shifted upward by 0.7 cm^−1^ from the wing region to the window regions, while downward by 0.3 cm^−^^1^ to the uncoalesced mask regions. This indicates that the stress in the window region decreases dramatically compared with the GaN template. The stress in the wing regions is also larger than in the window regions.

**Figure 7:**
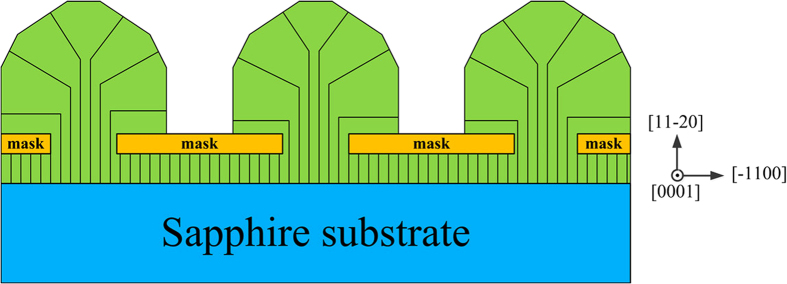
Schematic diagram of dislocation growth mechanism during GaN ELO process of sample B.

The obvious differences in Raman mapping results between two samples are due to the different dislocation growth mechanism during the ELO growth along different stripes. The variations in crystalline quality and stress distribution in different regions of sample B can be explained that when GaN grown along [0001] mask stripe direction, many TDs bend during the vertical growth. This phenomenon is explained by the dislocation following a path of minimum elastic energy per unit of growth length of materials^37^. As shown in Fig. 7, the density of dislocations reduces on the top of the window region as a result of many the dislocation lines bending from the window region into the wing region for stripes aligned along the [0001] direction, instead of propagating straightly to the top facets^28^. So the crystalline quality at the window region is much better. Besides, the thickness reduces from the wing-window boundary to the wing-mask boundary which could also lead to the intensity of the wing region is smaller than that in the window region^32^. The distinctive contrast in intensities and FWHM between the wing and window regions demonstrates that the E_2_ (high) mode is quite sensitive to spatial variations in crystalline quality. The decrease of dislocations density in the window regions may result in the reduce of residual stress^38^.

**Figure 8:**
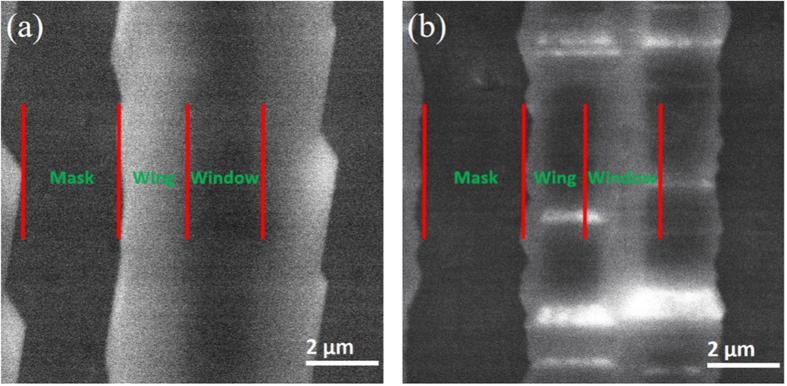
Plan-view monochromatic 366 nm CL mapping images of the two samples with electron acceleration voltages of 20 kV at 5000-time magnification: (**a**) sample A and (**b**) sample B.

In order to further investigate the spatial distributions of TDs in the two samples, SEM-CL studies have been carried out. Here, the region with higher density of TDs shows lower luminescence intensity which may be due to the TDs acting as nonradiative recombination centers^39^. Figure 8 illustrates the plan-view monochromatic 366 nm CL mapping images of the two samples with electron acceleration voltages of 20 kV at 5000-time magnification.

From Fig. 8(a,b), the mask regions and GaN stripes can be easily identified as the mask regions have the lowest luminescence intensity due to the highest dislocation intensity in the mask regions. As shown in Fig. 8(a), the width of the darker stripes in the GaN stripe regions is about 2 μm, in keeping with the width of the window region. This indicates that the density of the TDs in the window regions is higher. On the other hand, the wing regions are clearly brighter than other regions which indicates that the TDs density of the wing regions is much lower than the window regions in sample A. In contrast, it is obvious noticed there is a brighter stripe in the window region in Fig. 8(b). According to the SEM image in Fig. 2(b), this brighter stripe is corresponding to the top facet region of the GaN stripe. However, the other region of the GaN stripe is darker which confirms that the intensity of TDs is much higher. So it indicates that many dislocations in the window region bend to the wing region in sample B. The spatial distributions of dislocations obtained from the CL characterization could further support the dislocation growth mechanisms during the ELO processes in the two samples we propose according to the Raman mapping results.

## Conclusion

In summary, the spatial mapping of micro-Raman measurements have been performed on uncoalesced ELO GaN structures along SiN_x_ mask stripes both parallel to [0001] and 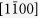. Due to the anisotropic in a-plane GaN, the spatial distribution of crystalline quality and strain in different regions of the GaN stripe is realized much discrepancy when the stripes are along different orientations. When the stripes are along [0001], the window regions have the highest intensity and least stress, which indicates the perfect crystalline quality and few dislocations of the window regions. In contrast, when the stripes are parallel to 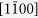, the quality of wing regions is much better than that of window region. This is probably due to the different dislocation bending preferences during the growth along different orientations. The bending of the dislocation could lead to the improvement of the crystal quality and reduction of residual stress. Spatial CL mapping results further support the interpretation of the dislocation growth mechanisms during the ELO processes with different mask stripe orientations.

## Additional Information

**How to cite this article**: Jiang, T. *et al.* Spatially resolved and orientation dependent Raman mapping of epitaxial lateral overgrowth nonpolar a-plane GaN on r-plane sapphire. *Sci. Rep.* **6**, 19955; doi: 10.1038/srep19955 (2016).

## Supplementary Material

Supplementary Information
