# Supplementary material for: Spatially resolved and orientation dependent Raman mapping of epitaxial lateral overgrowth nonpolar a-plane GaN on r-plane sapphire
Source: Sci Rep. 2016 Jan 29;6:19955. doi: 10.1038/srep19955 (PMC4731808; doi:10.1038/srep19955)
Supplement: Supplementary Information [file srep19955-s1.doc]

**Spatially resolved and orientation dependent Raman mapping of epitaxial lateral overgrowth nonpolar a-plane GaN on r-plane sapphire**

Teng Jiang^1^, Sheng-rui Xu^1^, Jin-cheng Zhang^1^, Yong Xie^2^, and Yue Hao^1^

^1^Wide Bandap Semiconductor Technology Disciplines State Key Laboratory, School of Microelectronics, Xidian University, Xi'an 710071, China, ^2^ School of Advanced Materials and Nanotechnology, Xidian University, Xi'an 710071, China.

To further support the Raman studies for uncoalesced GaN stripes, a spatial mapping scan of the E_2_ (high) mode have been measured to the coalesced GaN layer obtained from the regrowth of sample A. Figure 1 shows the Raman mapping spectrum of the intensities, the full width at half maximums (FWHMs) and peak positions of E_2_ (high) modes of the coalesced GaN layer. Similar to Figure 4 in the manuscript, the density in the window regions is lower than that in the wing region while both the FWHM and the peak position in the wing region are smaller than that in the wing region. This further improves that the wing regions have better crystalline quality and less residual stress.


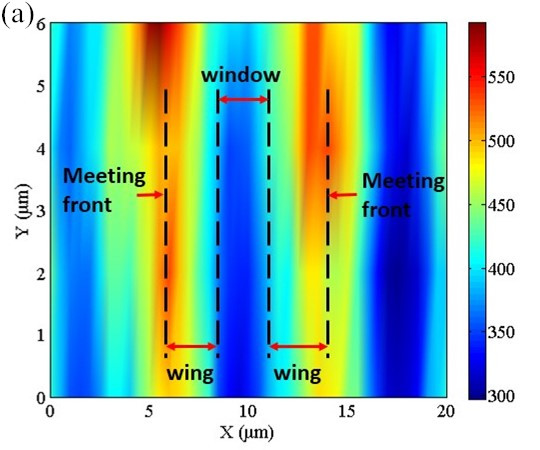

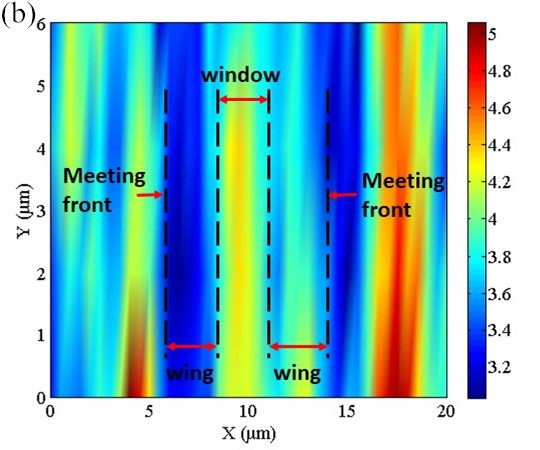


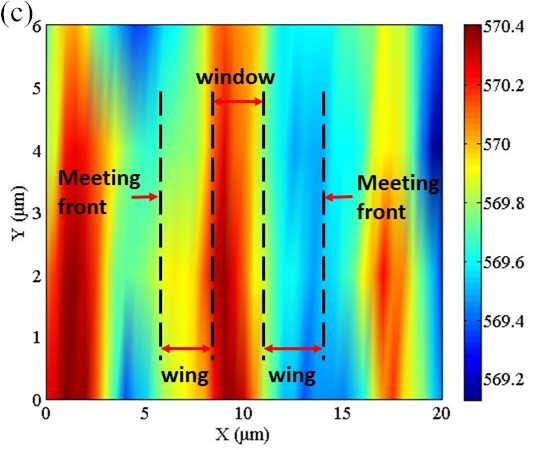


**Figure 1** | (a) Raman mapping spectrum of E_2_ (high) mode intensity of coalescent sample A; (b) Raman mapping spectrum of E_2_ (high) mode FWHM of coalescent sample A; (c) Raman mapping spectrum of E_2_ (high) mode peak position of coalescent sample A.
